# Decoding the chicken gastrointestinal microbiome

**DOI:** 10.1186/s12866-024-03690-x

**Published:** 2025-01-20

**Authors:** PB. Burrows, F. Godoy-Santos, K. Lawther, A. Richmond, N. Corcionivoschi, SA. Huws

**Affiliations:** 1 School of Biological Sciences, Institute for Global Food Security, Queen’s University Belfast, 19 Chlorine Gardens, Belfast, BT9 5DL UK; 2 Moy Park, Food Park, 39 Seagoe Office, Portadown, BT63 5QE Craigavon UK; 3 Bacteriology Branch, Veterinary Sciences Division, Agri-Food and Biosciences Institute, 12 Stoney Road, Belfast, BT4 3SD UK; 4 Faculty of Bioengineering of Animal Resources, University of Life Sciences King Mihai I from Timisoara, Timisoara, 300645 Romania; 5 Academy of Romanian Scientists, Ilfov Street, No. 3, Bucharest, 050044 Romania

**Keywords:** Broiler, GIT sites, Microbiota, 16S rRNA gene, Metataxonomy, Gut, Diversity, Core microbiome

## Abstract

Metataxonomic studies have underpinned a vast understanding of microbial communities residing within livestock gastrointestinal tracts, albeit studies have often not been combined to provide a global census. Consequently, in this study we characterised the overall and common ‘core’ chicken microbiota associated with the gastrointestinal tract (GIT), whilst assessing the effects of GIT site, bird breed, age and geographical location on the GIT resident microbes using metataxonomic data compiled from studies completed across the world. Specifically, bacterial 16S ribosomal DNA sequences from GIT samples associated with various breeds, differing in age, GIT sites (caecum, faeces, ileum and jejunum) and geographical location were obtained from the Sequence Read Archive and analysed using the MGnify pipeline. Metataxonomic profiles produced across the 602 datasets illustrated the presence of 3 phyla, 25 families and 30 genera, of which core genera (defined by presence in over 90% of datasets) belonged to *Lactobacillus*, *Faecalibacterium*, *Butyricicoccus*, *Eisenbergiella*, *Subdoligranulum*, *Oscillibacter*, *Clostridium* & *Blautia*. PERMANOVA analysis also showed that GIT site, bird breed, age and geographical location all had a significant effect on GIT microbial diversity, regardless of dietary factors, which were not considered in this study. On a genus level, *Faecalibacterium* was most abundant in the caeca, *Lactobacillus* was most abundant in the faeces, ileum and jejunum, with the data showing that the caeca and faeces were most diverse. AIL F8 progeny, Ross 308 and Cobb 500 breeds GIT bacteria were dominated by *Lactobacillus*, and *Eisenbergiella*, *Megamonas* and *Bacteroides* were most abundant amongst Sasso-T451A and Tibetan chicken breeds. Microbial communities within each GIT site develop with age, from a *Lactobacillus* and *Streptococcus* dominated community during the earlier stages of growth, towards a *Faecalibacterium*, *Eisenbergiella*, *Bacteroides*, *Megamonas*, and *Lactobacillus* dominated community during the later stages of life. Geographical locations, and thus environmental effectors, also impacted upon gastrointestinal tract microbiota, with Canadian and European datasets being dominated by *Lactobacillus*, whilst UK and Chinese datasets were dominated by *Eisenbergiella* and *Bacteroides* respectively. This study aids in defining what ‘normal’ is within poultry gastrointestinal tract microbiota globally, which is imperative to enhancing the microbiome for productive and environmental improvements.

**Supplementary Information:**

The online version contains supplementary material available at 10.1186/s12866-024-03690-x.

## Introduction

The domesticated broiler chicken, *Gallus gallus domesticus*, is amongst the leading global food source, attributed to the rich protein content and micronutrients, coupled with low production cost of broiler chicken [1]. Indeed, broiler chicken production has reached an impressive scale of 74 billion chicken slaughtered globally [2], with expectations of reaching 121% of production rates by 2050 as compared with 2005 levels [3]. In this context, it is vital to recognise the importance of sustainably enhancing chicken production in order to address the challenges of accommodating the globally increasing demand [4–6]. The productive capabilities of chicken are affected by many factors related to the host (genetics, immune response, general gut health) and environment (farm management, chicken welfare, feed), which all have a direct impact on the gastrointestinal tract (GIT) microbiota [7]. Simultaneously, GIT microbiota significantly influence the health and metabolism of broilers [8], highlighting the importance of having a comprehensive understanding of GIT associated microbiomes in order to sustainably enhance broiler chicken production.

Poultry are monogastric animals with a sophisticated physiology, whereby the digestive system consists of the crop, proventriculus, ventriculus (gizzard), small intestine (duodenum, jejunum, ileum), large intestine, caeca, colon and cloaca. Proximal gut (crop to ventriculus) is highly involved with softening feed through enzymatic and mechanical breakdown, within an acidic environment [9]. The distal gut (small intestine, caeca and colon) further extracts nutrients from digested feed and non-starch polysaccharides (NSP) present in the feed [9]. The complex microbial ecosystems within the chicken’s GIT are often dominated by lactic acid bacteria within the proximal gut, including the genera *Lactobacillus*, *Bifidobacterium*, *Enterobacteriaceae* and *Klebsiella* which initiate the digestion of feed [10–12]; whereas *Lactobacillus*, *Enterococcus*, *Clostridia*, *Streptococcus*, *Bacteroides*, coliforms, *Faecalibacterium*, *Ruminococcus* are often abundant within the distal gut microbial communities [11–16].

A bacterial census of poultry intestinal microbiome, published in 2013, utilized data from past metataxonomic studies deposited in three public databases: GenBank, SILVA comprehensive ribosomal RNA database, and Ribosomal Database Project which were made available through the MG-RAST server (https://www.mg-rast.org/mgmain.html?mgpage=search&search=Poultry_Gut_DB = search&search = Poultry_Gut_DB) [17]. In that study a total of 3,184 16S rRNA gene sequences were characterized obtained using various primers that targeted different hypervariable regions of the 16S rRNA gene. These sequences were sourced from chicken caeca and from intestinal samples (without specification of the local intestine section used such as duodenum, ileum or jejunum). That study identified 12 phyla from intestinal sequences, whereby Firmicutes dominated (70%), followed by Bacteroidetes (12.3%) and Proteobacteria (9.3%); meanwhile 10 phyla were identified from the caecal data, where again, Firmicutes (78%) and Bacteroidetes (11%) were the dominating phyla. Intestinal sequences highlighted the presence of numerous genera belonging to Firmicutes accounting for > 1% of total sequences (*Clostridium*, *Ruminococcus*, *Lactobacillus*, *Eubacterium*, *Fecalibacterium*, *Butyrivibrio*, *Ethanoligenens*, *Alkaliphilus*, *Butyricicoccus*, *Blautia*, *Hespellia*, *Roseburia*, & *Megamonas*) [17]; likewise Bacteroidetes were represented by four genera (*Bacteroides*, *Prevotella*, *Parabacteroides* & *Alistipes*) composing > 1% of total sequences. With respect to other phyla, only one genus from Actinobacteria (*Bifidobacterium*) and Proteobacteria (*Desulfohalobium*) presented a notable abundance within the intestinal microbiome at > 1% and 0.7% of total sequences respectively [17]. Amongst the caecal samples, 31 genera belonged to Firmicutes, of which three represent > 5% of read abundance (*Ruminococcus*, *Clostridium* & *Eubacterium*), and ten represented > 1% of read abundances (*Fecalibacterium*, *Blautia*, *Butyrivibrio*, *Lactobacillus*, *Megamonas*, *Roseburia*, *Ethanoligenes*, *Hespellia*, *Veillonella*, & *Anaerostipes*). *Bacteroides* was the most abundant of the Bacteroidetes phylum accounting for 4% of total caecal sequences with other genera present (*Prevotella*, *Paraprevotella*, *Tanneralla* and *Riemeralla*). Proteobacteria were low in abundance and represented mainly by three genera (*Desulfohalobium*, *Escherichia*/*Shigella* & *Neisseria*) [17]. In addition, Chica Cardenas et al. 2021 [18] performed a similar study in 2021, producing a meta-analysis of chicken caeca microbial communities using data targeting the V3, V4 and V3-V4 hypervariable regions from 9 studies accounting for 324 total samples [18]. Upon comparing each of the hypervariable regions, they identified *Oscillospira* amongst all 3 using an 80% abundance cut-off, after the cut-off was reduced to 50%, 5 genera (*Oscillospira*, *Lactobacillus*, *Faecalibacterium*, *Clostridium* and *Ruminococcus*) were identified [18]. Overall, the selection of hypervariable regions, in relation to assessing metataxonomic data, affects the evaluation of microbiomes and Chica Cardenas et al. 2021 [18] found hypervariable region V4 presents the most diverse and most unique genera when compared with the other regions.

Given that only two poultry GIT microbiota census were available [17, 18], with Wei et al. 2013 [17] utilising sequences from different variable regions, which in itself has been shown to be a variable affecting bacterial metataxonomic results, and Chica Cardenas et al. 2021 [18] focusing systematically on caecal data, resulting in only 324 sequence datasets being included in their analysis, it is now timely to re-visit the concept of the core poultry microbiome and factors which affect GIT bacterial colonisation using more comprehensive data. It should also be noted that neither study investigated the effect of geographical and GIT site or bird age either, although Chica Cardenas et al. 2021 [18] did investigate breed and noted that there was a breed effect on the caecal bacteria present. Therefore, our aim in this study is to provide a comprehensive up to date study of our current understanding of the composition and diversity of chicken GIT microbiomes and factors which control this (GIT site, breed, bird age and geographical location) using all publicly available 16S rRNA gene sequences targeting the V3-V4 hypervariable regions only, allowing an enhanced understanding of these microbiomes and factors which effect their development in the chicken GIT on a global scale.

## Methods and materials

### Selection of sequence read archive data and bioprojects

Sequence read archive (SRA) data relating to chicken GIT 16S rRNA gene sequences were selected from the SRA database between the months of February and July 2020, whilst using and combining search terms “chicken”, “broiler”, “hen”, “gastrointestinal tract”, “GIT”, “microbiome”, “microbiota”, “caecum”, “intestine” and “faeces”. Bioprojects containing substantial associated metadata were selected and excluding studies exhibiting any additional factors, such as intentional infection with *Campylobacter* spp., were excluded to prevent interference or bias when analysing the core microbiota, although any control treatments were taken into consideration. A total of 114 bioprojects were selected pertaining to 6,742 individual sequencing datasets. These bioprojects were further refined by removing those which excluded important information such as GIT site; age of birds; breed and geographical location. Subsequently, only bioprojects which were obtained using the 16S rRNA gene V3-V4 hypervariable regions were chosen as this was the most common hypervariable regions analysed. The choice of using data obtained using the same primers targeting the V3-V4 hypervariable region was in order to reduce the non-biological variability in the results. After refining our initially downloaded 114 bioprojects, 11 bioprojects were selected, of which 602 sequencing datasets were identified. The full metadata collected from these sequence datasets can be found in Supplementary File (1) Microsoft Excel Document. A summary breakdown of the studies involved can be found in Supplementary Tables 1 and Supplementary Table (2). Subsequently, the 11 bioprojects datasets were submitted to the MGnify pipeline (https://www.ebi.ac.uk/metagenomics) for analysis [19].

### Data processing

Outputs from the MGnify pipeline contained OTU count data at each taxon level; phylum, family and genus (Supplementary File 1. Microsoft Excel Document). Relative abundances were calculated by the application of the Total Sum Scaling (TSS) method using the following formula: OTU read count divided by (the total OTU read counts of a sample divided by the minimum total OTU read counts across the dataset). Relative abundances were calculated at phylum, family and genus taxon levels. OTU abundances less than 95% were categorised as ‘Other’. Relative abundances were subsequently grouped into GIT site (caecum, faeces and small intestine (jejunum and ileum)). Within each GIT site, data was grouped pertaining to each parameter (GIT site, breed, bird age and geographic location) to identify the prominent taxa in each parameter along with common core community members.

### Computational statistical analysis

Data was tabulated into bar and pie charts using Excel, and Principal Component Analysis (PCA) plots were plotted using R (ver. 4.3.2). Venn diagrams were produced based on the common core microbiome across data groups using the website http://www.interactivenn.net/index.html [20]. Alpha and Beta diversity measures were determined using ‘vegan’ in R (ver. 2.6-4) [21]. Scripts can be found in Supplementary File 2 R Script Document. The effect of each parameter, as well as their interactions on OTU read counts, was assessed using permutational multivariate analysis of variance (PERMANOVA). This analysis was based on Bray-Curtis dissimilarity and was conducted via the ‘adonis2’ function in the vegan package, employing 1000 permutations. Normalised data through a variance-stabilising transformation (VST) implemented by DESeq2 (version 1.42.0) [22] was visualised using PCA plots.

## Results

### Summary of chicken GIT bacterial microbiome

**Fig. 1:**
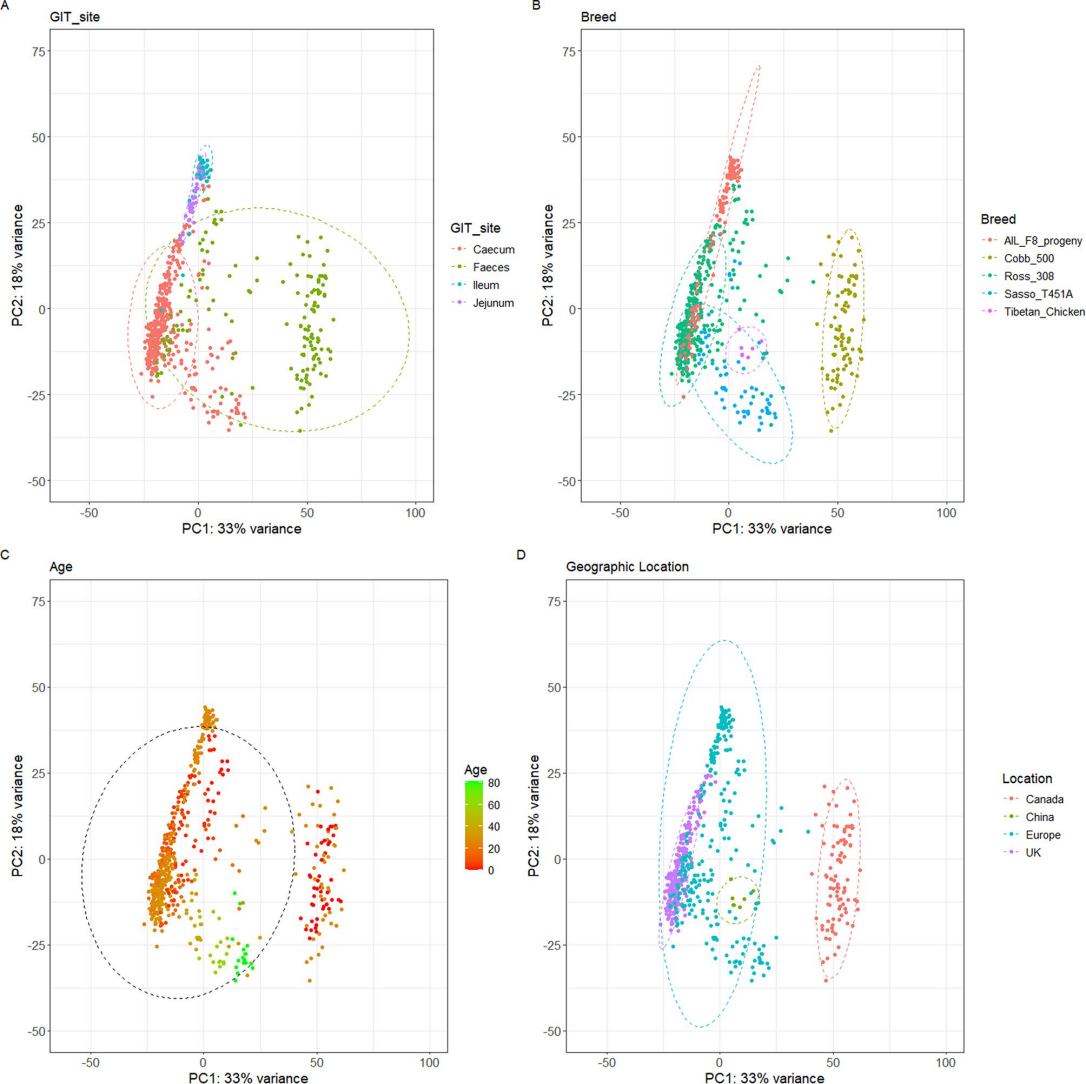
Principle component analysis of OTU read counts of all datasets at genus level. The PCA plots highlight the variability in OTU read counts subsequent to normalisation using variance-stabilising transformation (VST). The plots are organised to present: (**A**). GIT site; (**B**). bird breed; (**C**). bird age; (**D**). geographical location. Principle component 1 explains 33% variance, as principle component 2 highlights 18% variance

Principle component analysis (PCA) of normalised OTU read counts demonstrate clustering of communities according to each variable examined (Fig. 1). Regardless of variable, the principle component 1 and 2 explained 33% and 18% variance respectively. The communities derived from different GIT sites (caecum, ileum and jejunum) resemble each other and cluster together, the caecum is most spatially deviated, although with a densely clustered focal point separated from the ileum and jejunum, which in turn are compressed clusters presenting similarity (Fig. 1A). However, the faecal communities are separately clustered, with some deviating to resemble the caecum (Fig. 1A). GIT OTUs across breeds Ross 308, AIL F8 and Sasso T451A also cluster together, whilst Cobb 500 OTUs are distinctly different from the other breeds and cluster away from the other sample OTUs (Fig. 1B & C). Likewise, when comparing geographical locations, the European, UK and China-derived chicken GIT samples present most similarity, with OTUs generated from the GIT of Canadian clustering separately, indicating different taxonomic diversity within (Fig. 1D).

**Fig. 2:**
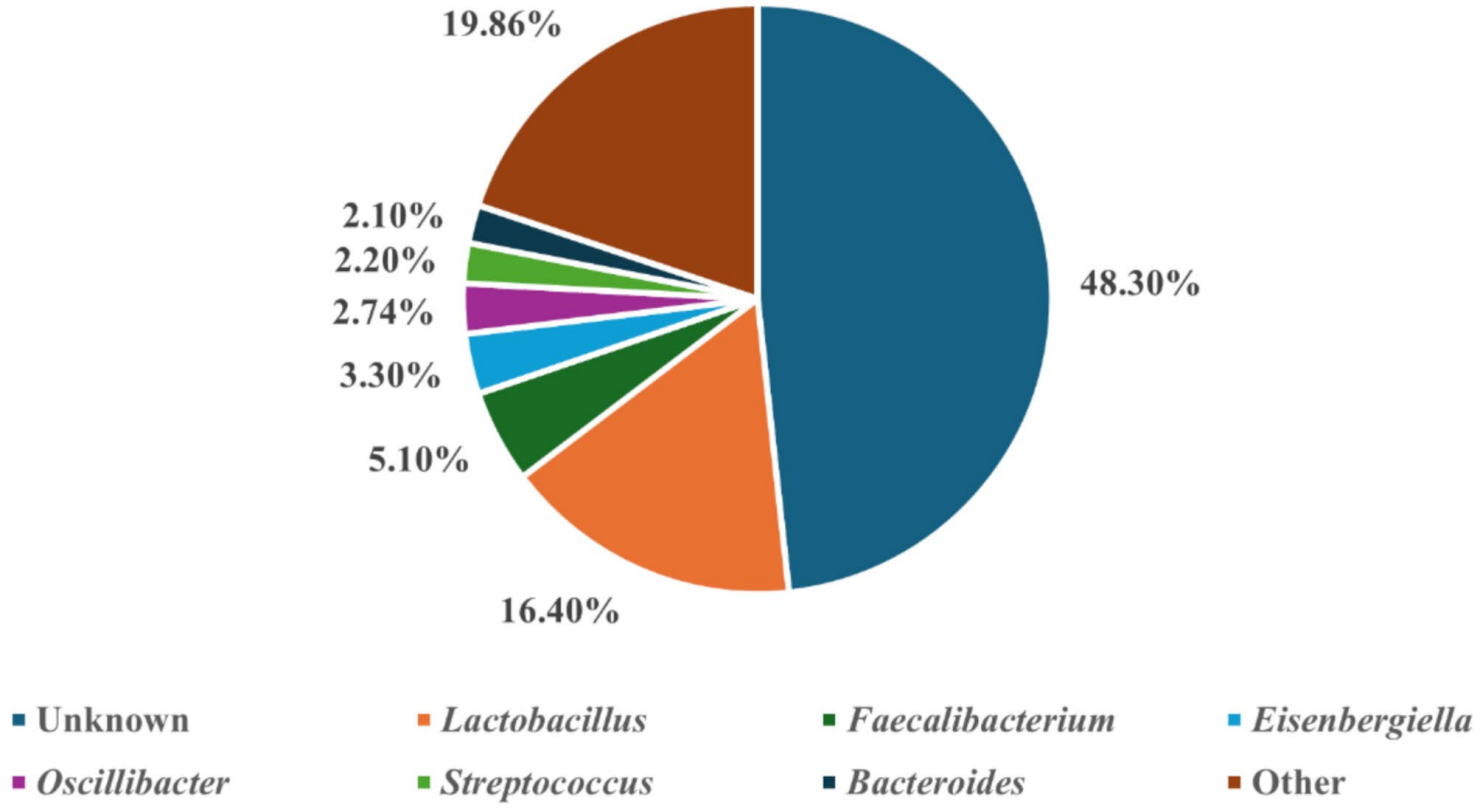
Relative abundances of total datasets at genus level. Relative abundances of total datasets highlight 48.30% were of unknown genus with the remaining 51.70% as being identified. 31.84% relative abundance of which were labelled above whilst the remaining 19.86% consists of 687 genera comprising of > 2%. Pie chart was produced in Microsoft Excel. Pie charts of the total relative abundances at phylum and family level can be found in Supplementary File 1. Microsoft Excel Document

A total of 65,186,954 OTU read counts were identified across all datasets ranging from 1,385 to 755,749. Median read counts that been taxonomically classified were 91,383 for caecal, 102,173 for faecal, 4,463 for jejunal and 53,040 for ileal. By way of taxonomic assignment, 99.02% (64,547,963 reads) OTUs were classified to the Bacteria domain, as 0.98% (638,991 reads) were classified to the Archaea domain. Amongst the top 95% of sequences across all 602 datasets available, we found three phyla, twenty-three family and twenty-eight genera (Supplementary File 1. Microsoft Excel Document). Dominant phyla include: Firmicutes, Proteobacteria and Bacteroidetes, accounting for 80.62%, 7.89% and 5.91% of total read abundances respectively. On a family level *Ruminococcaceae*, *Lactobacillaceae* and *Lachnospiraceae* were the most abundant, accounting for 23.10%, 16.53% and 10.80% of read abundances respectively. Ten families ranged between 5% and > 1% of sequence read abundances, of these the most abundant were *Enterobacteriaceae*, *Oscillospiraceae* and *Clostridiaceae*, accounting for 4.87%, 2.76% and 2.53% respectively. On a genus level, *Lactobacillus*, *Faecalibacterium*, *Eisenbergiella* and *Oscillibacter* were the most abundant, accounting for 16.40%, 5.10%, 3.30% and 2.74% of total read abundances respectively (Fig. 2), and eight other genera represented > 1% of sequence read abundances (*Streptococcus*, *Bacteroides*, *Butyricicoccus*, *Alistipes*, *Enterococcus*, *Megamonas*, *Ruminiclostridium* and *Romboutsia*).

PERMANOVA analyses identified significant differences in the microbial communities based on GIT site, breed, geographical location and age (*P* < 0.001). GIT site as a variable was identified as the main contributor affecting the microbiome, followed by breed and age. In contrast, geographic location had a comparatively smaller effect (*P* < 0.001) (Table 1). Similarly, ANOVA analyses of alpha diversity indices revealed that GIT sites consistently explained the largest proportion of variation across all indices (*P *≤ 0.001) (Table 2). While bird age showed significant differences across all indices when compared with breed, the interaction terms revealed more specific patterns. GIT site and age interactions were not significant for any diversity index, whereas the interaction between geographical location and age was significant only for Inverse Simpson diversity (*P* = 0.000199).

**Table 1:** Permutational multivariate analysis of variance (PERMANOVA) analysis of OTU read counts after TSS scaling to evaluate effects of independent factors including GIT site, bird breed, bird age and geographical location at genus level. PERMANOVA highlights significant differences between each of the independent variables (*P* < 0.001)

	*R* ^2^	F	*P*-value	
GIT site	0.3894	277.4005	1.00E-04	***
Breed	0.1576	84.2206	1.00E-04	***
Geographical location	0.0198	42.3992	1.00E-04	***
Age	0.1509	8.2701	1.00E-04	***
GIT site: Age	0.0167	3.9654	1.00E-04	***
Breed: Age	0.0009	1.8415	0.08849	.
Geographical location: Age	0.0111	11.8616	1.00E-04	***

The codes ‘*’, ‘**’, ‘***’, denotes levels of significance (0.05, 0.01 & 0.001 respectively) in differences amongst factors

**Table 2:** Analysis of variance (ANOVA) of alpha diversity indices using OTU read counts after TSS scaling to evaluate effects of independent factors including GIT site, bird breed, bird age and geographical location at genus level. ANOVA was performed on alpha diversity indices including Chao1 richness, Pielou’s evenness, Shannon and Inverse Simpson estimating the impact each independent factor of GIT site, breed, location and age have on tested indices. The codes ‘*’, ‘**’, ‘***’, denotes levels of significance (0.05, 0.01 & 0.001 respectively) in differences amongst factors

	Chao1			Evenness			Shannon			Inverse Simpson		
	F	*P*-value		F	*P*-value		F	*P*-value		F	*P*-value	
GIT site	603.065	2.00E-16	***	176.491	2.00E-16	***	187.380	2.00E-16	***	86.605	2.00E-16	***
Breed	121.110	2.00E-16	***	42.473	2.00E-16	***	65.196	2.00E-16	***	35.852	2.00E-16	***
Geographical location	10.523	0.001	**	31.246	3.48E-08	***	40.382	4.19E-10	***	22.243	3.00E-06	***
Age	73.244	2.00E-16	***	30.282	5.58E-08	***	74.332	2.00E-16	***	56.381	2.23E-13	***
GIT site: Age	3.484	0.062	.	0.189	0.664		0.742	0.389		0.021	0.885	
Breed: Age	74.956	2.00E-16	***	18.479	1.65E-08	***	35.817	2.10E-15	***	26.604	8.70E-12	***
Geographical location: Age	2.265	0.133		0.632	0.427		1.951	0.163		14.016	0.000199	***

Codes: 0 ‘***’ 0.001 ‘**’ 0.01 ‘*’ 0.05 ‘.’ 0.1 ‘ ’ 1

### Effect of gastrointestinal tract site on the microbial diversity

**Fig. 3:**
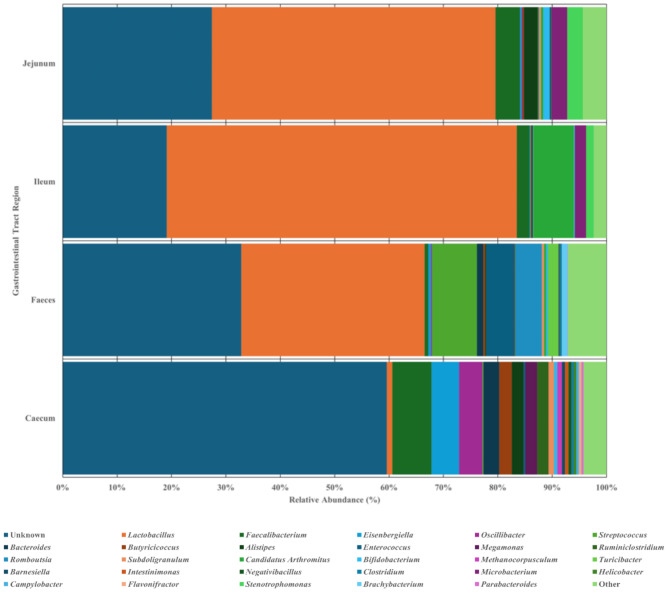
Microbial communities of each GIT site at genus level. Relative abundance bar chart showing the abundances of genera within caecal, faecal, ileal and jejunal samples. Up to 59.58% of genera were classfied as unknown in caecal samples, while up to 32.83% were classified as unknown in other sample types, where *Lactobacillus* dominate reaching 64.35%. Relative abundance graphs have been produced using normalised data presenting abundances across all datasets but grouped into each variable

Caecal and faecal samples exhibited the highest diversity among the samples associated to GIT, with the faecal samples being the most species rich. In contrast, the small intestine sections showed comparable values across measures of richness, evenness, Shannon and Inverse Simpson indices (*P* < 0.001; Supplementary Fig. 1; Table 2). Following grouping into associated GIT sites, 375 datasets were associated with the caecum, and were composed of three phyla, fourteen families and twenty genera based on the top 95% of reads; 152 datasets were obtained for faeces, and were composed of four phyla, eighteen families and twenty genera; 38 datasets were obtained for the ileum, and were composed of two phyla, nine family and four genera; 37 datasets were obtained for the jejunum, and were composed of three phyla, thirteen family and seven genera. At the genus level, abundances across each GIT site vary substantially (Fig. 3), whereas *Faecalibacterium* is most abundant in the caecum followed by *Eisenbergiella* and *Oscillibacter* (7.22%, 5.07% & 4.30% of total read abundances respectively); meanwhile *Streptococcus* and *Enterococcus* follow *Lactobacillus* in terms of faecal sample dominance (8.27% & 5.05% of total read abundances respectively). This suggests there is little similarity between caecal and faecal samples. Ileal and jejunal datasets present slightly different abundances; following *Lactobacillus* in abundance were *Candidatus arthromitus* and *Faecalibacterium* (7.40% & 2.37% of total read abundances respectively) in ileal samples, while *Faecalibacterium* and *Stenotrophomonas* (4.53% & 2.88% of total read abundances respectively) follow in abundance in jejunal samples. Data and information regarding each taxonomic level can be found in Supplementary File 1. Microsoft Excel Document.

### Effects of breed on the gastrointestinal tract microbial diversity

**Fig. 4:**
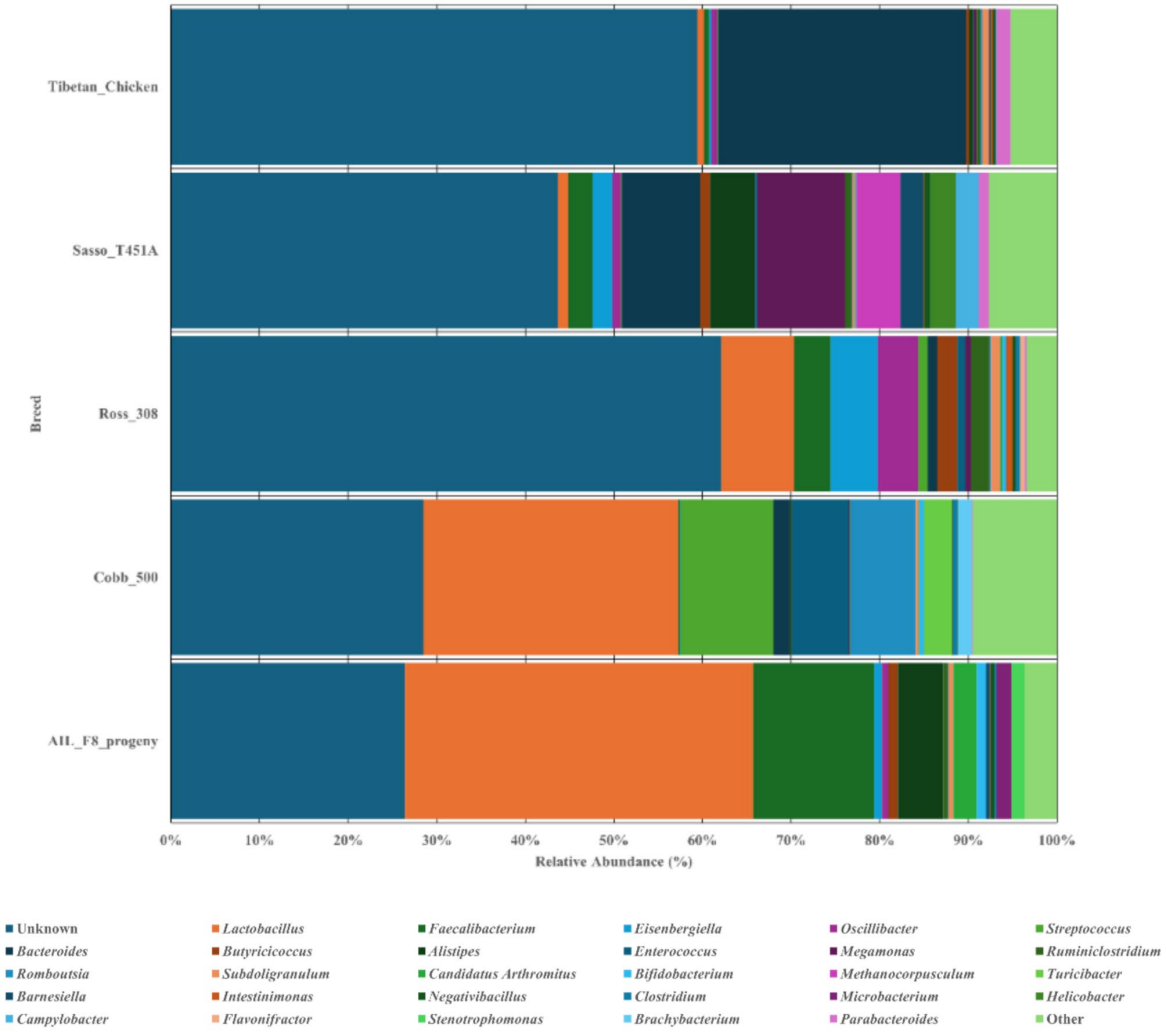
Microbial communities of each breed at genus level. Relative abundance bar chart showing the abundances of genera within each breed (AIL F8, Cobb 500, Ross 308, Sasso T451A and Tibetan chicken samples). 26.40 − 62.10% of genera were classified as unknown across all variables, where *Lactobacillus* dominate AIL F8, Ross 308 and Cobb 500 reaching 39.35% of total relative abundance, and *Bacteroides* dominates Tibetan chicken with 28.08% of total relative abundances. Sasso T451A is dominated by *Megamonas* and *Bacteroides* (9.91% & 8.89%) respectively. Relative abundance graphs have been produced using scaled data presenting abundances across all datasets but grouped into each variable

Five breeds were identified across all datasets, all have a high abundance of unknown genera from 26.40% (AIL F8) to 62.10% (Ross 308) (Fig. 4). *Lactobacillus* is most abundant of the known genera in the breeds AIL F8, Cobb 500 and Ross 308. *Lactobacillus* was followed by *Faecalibacterium* in AIL F8 (39.35% & 13.65% of total read abundances respectively). Cobb 500 highlights *Lactobacillus* (28.79% of total read abundances) and *Streptococcus* (10.54% of total read abundances) as most abundant respectively, accounting for 39.32% of total relative abundances. Likewise, *Lactobacillus* is followed by *Eisenbergiella* amongst Ross 308 (8.25% & 5.32% of total relative abundances respectively). Sasso T451A presents *Megamonas* and *Bacteroides* as most abundant (9.91% & 8.89% of total relative abundances respectively). Meanwhile, *Bacteroides* and *Parabacteroides* were the most abundant genera of the Tibetan breeds (28.08% & 1.62% of total relative abundances). In terms of diversity, all estimated diversity indices were significantly different (*P* < 0.001), Cobb 500, Ross 308 and Sasso T451A were amongst the most diverse, as Tibetan chicken breeds follow. Cobb 500 is most rich, as Ross 308 and Sasso T451A were most even (Supplementary Fig. 2; Table 2).

### Effect of bird age on the gastrointestinal tract microbial diversity

**Fig. 5:**
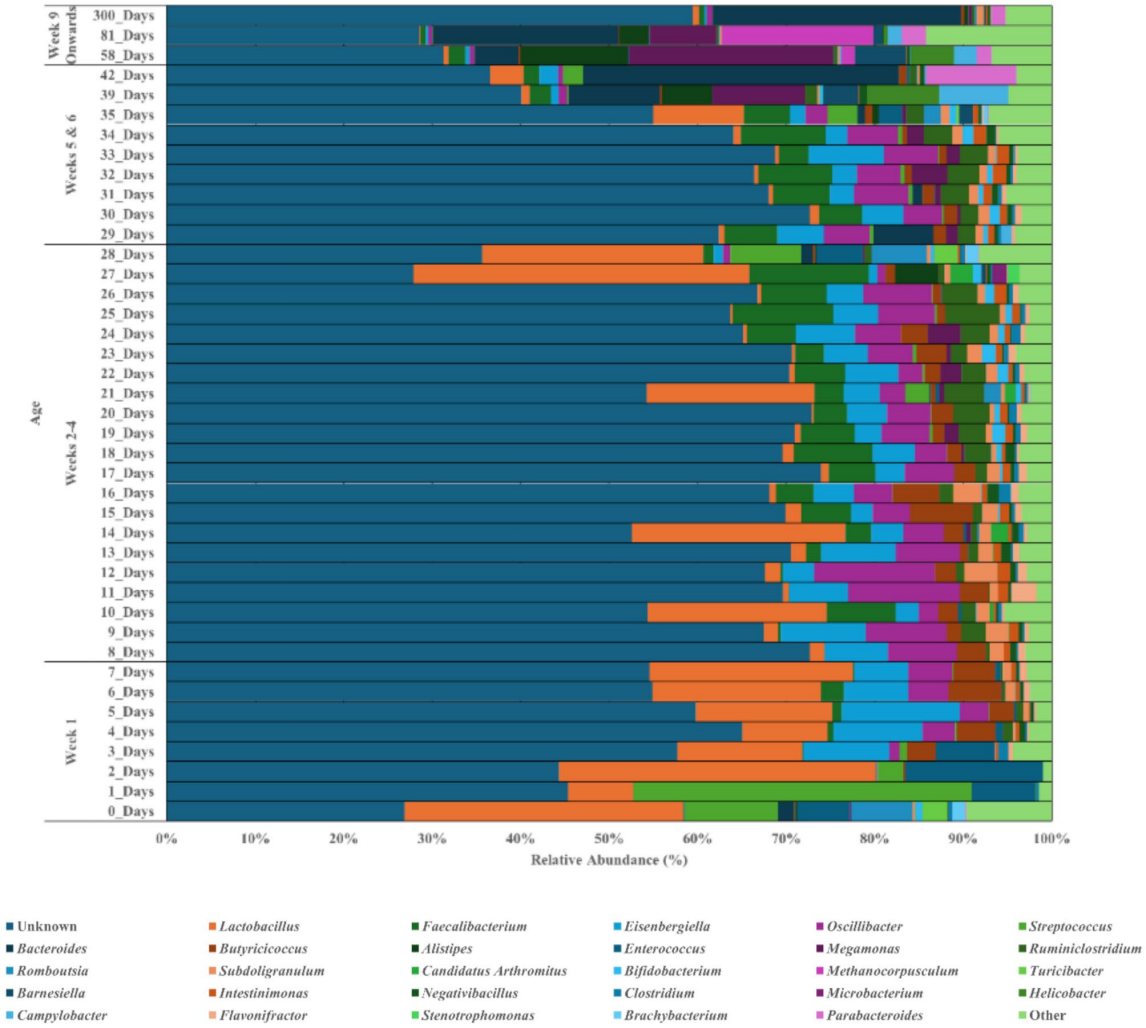
Microbial communities of each age range at genus level. Relative abundance bar chart showing the abundances of genera during weeks 1 to beyond week 9. 26.87 − 73.94% of genera were classified as unknown across all variables, where *Lactobacillus* is amongst the most abundant during the earlier ages reaching 35.80%, where *Faecalibacterium*, *Eisenbergiella* and *Oscillibacter* were amongst the dominating genera from the earlier ages to approaching day 35 (reaching 13.47%, 9.62% and 13.65% respectively). Relative abundance graphs have been produced using scaled data presenting abundances across all datasets but grouped into each variable

Datasets relating to forty-one separate ages were grouped together as follows: Week 1: 0–7 days old (130 datasets), Week 2: 8–14 days old (45 datasets), Week 3: 15–21 days old (78 datasets), Week 4: 22–28 days old (227 datasets), Week 5: 29–35 days old (64 datasets), Week 6: 39–42 days old (18 datasets) and Week 9 onwards: 58-‘>300’ days old (40 datasets) (Fig. 5). Week 9 onwards contains the dataset produced by Zhou et al. 2016 [23] who identified the Tibetan breeds as being older than 300 days.

During week 1, *Lactobacillus* dominated (40.72% of total relative abundances), followed by *Streptococcus* (10.19% of total relative abundances) and *Eisenbergiella* (10.13% of total relative abundances). There were minimal localised differences between days; on days 3, 5, 6 and 7, *Eisenbergiella* follows *Lactobacillus* in terms of abundance (9.72%, 13.32%, 7.23% & 6.12% of total relative abundances) but conversely, *Eisenbergiella* is more abundant than *Lactobacillus* on day 4 (9.63% of total relative abundances). On week 2, *Eisenbergiella* and *Oscillibacter* together dominate except for days 10 and 14, whereby *Lactobacillus* dominates (20.23% & 24.20% of total relative abundances). In terms of week 3 bacterial GIT diversity, *Butyricicoccus* is most abundant during days 15 and 16 (7.09% & 5.23% of total relative abundances), *Faecalibacterium* during days 18 and 19 (8.88% & 6.08% of total relative abundances), *Oscillibacter* during days 17 and 20 (5.59% & 4.95% of total relative abundances), whilst *Lactobacillus* dominates during day 21 (18.96% of total relative abundances). However, in week 3 as whole *Eisenbergiella*, *Oscillibacter* and *Faecalibacterium* were the most abundant genera, developing to *Eisenbergiella*, *Oscillibacter* and *Lactobacillus* dominated bacterial community during week 4. Days 22 and 24 were dominated by *Eisenbergiella* (5.99% & 6.74% of total relative abundances), whilst *Oscillibacter* dominates days 23, 25 and 26 (5.12%, 6.41% & 7.76% of total relative abundances), and *Lactobacillus* dominates on days 27 and 28 (37.08% & 24.98% of total relative abundances). By week 5, *Lactobacillus* is no longer amongst the dominating genera, except for day 35 where it is most abundant (10.22% of total relative abundances). A mix of *Faecalibacterium*, *Eisenbergiella*, *Bacteroides* and *Oscillibacter* were the most abundant during this week. *Faecalibacterium* dominates during days 30, 31, 32 and 34 (4.88%, 6.43%, 8.35% & 9.56% of total relative abundances); *Bacteroides* dominates day 29 (6.79% of total relative abundances) followed by *Faecalibacterium* (5.97% of total relative abundances), *Eisenbergiella* dominates during day 33 (8.52% of total relative abundances). These four genera comprise 13.88–23.20% relative abundances during this week. From week 6 onwards, abundant genera shift occur with *Megamonas* and *Bacteroides* being the dominating genera during day 39 (10.50% & 10.33% of total relative abundances), followed by *Helicobacter* and *Campylobacter* (8.17% & 7.83% of total relative abundances). Whereby by day 42, *Bacteroides* and *Parabacteroides* were the domaining genera (35.74% & 10.23% of total relative abundances). In week 9, day 58 is dominated *Megamonas* (23.10% of total relative abundances), while days 81 and > 300 were both dominated by *Bacteroides* (20.99% & 28.08% of total relative abundances), each followed by *Alistipes* (12.15% of total relative abundances), *Methanocorpusculum* (17.09% of total relative abundances) and *Parabacteroides* (1.62% of total relative abundances) respectively. These three dominating genera account for 35.25%, 38.08% & 29.71% of each age abundance respectively. Therefore, it can be concluded that as bird age, the community appears to shift away from *Lactobacillus* dominated as diversity increases (*P* < 0.001) (Supplementary Fig. 3; Table 2).

### Geographical location as an effect on the gastrointestinal tract microbiota

**Fig. 6:**
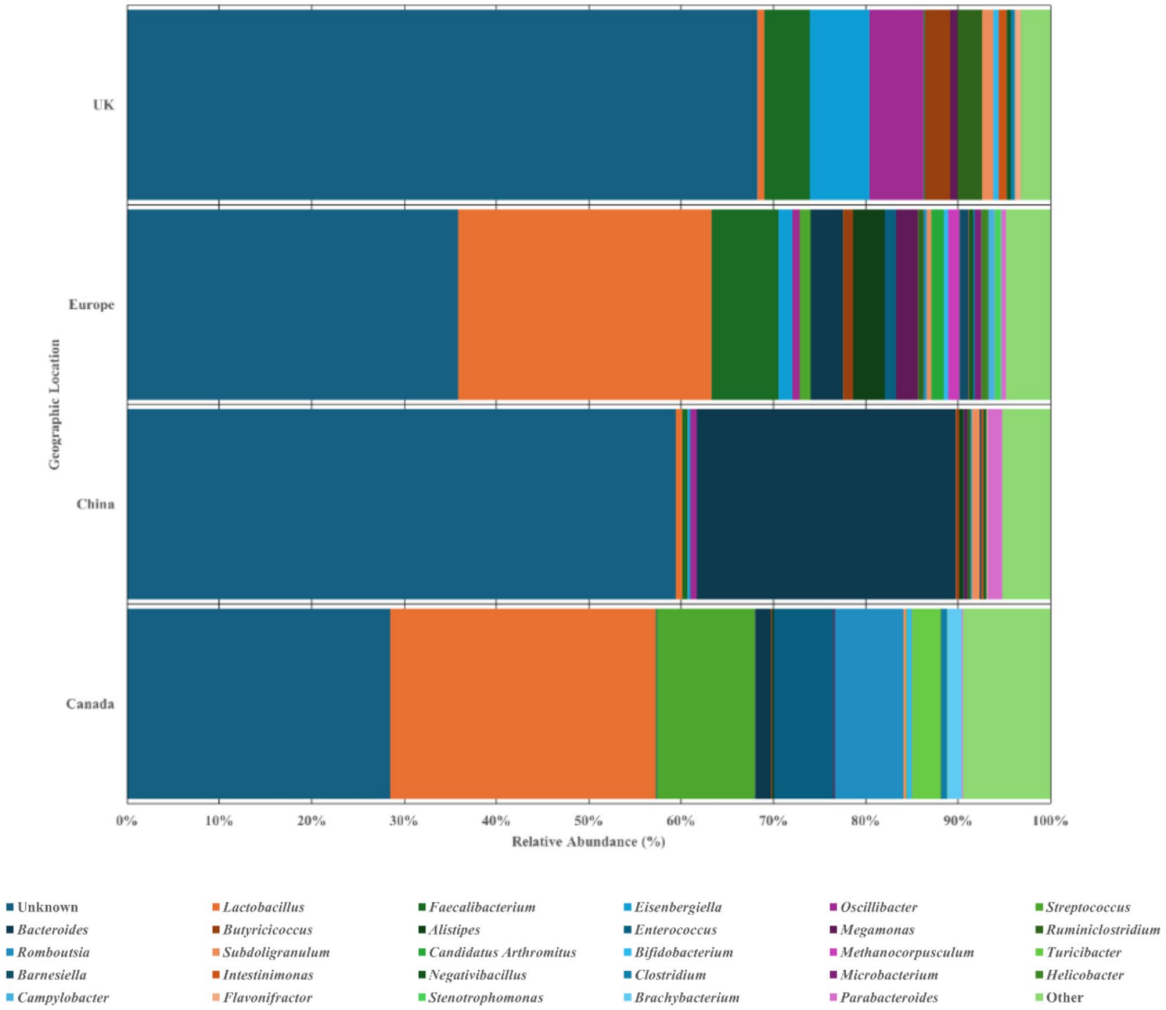
Microbial communities of each geographical location at genus level. Relative abundance bar chart showing the abundances of genera within the Canadian, Chinese, European and UK datasets. Genera classified as unknown ranged between 28.48 − 68.29% across all variables, where *Lactobacillus* dominate the Canadian and European datasets reaching 28.79%, and *Bacteroides* dominates Chinese datasets with 28.08%; *Eisenbergiella*, *Oscillibacter* and *Faecalibacterium* were amongst the most abundant in the UK datasets with 6.46%, 5.84% and 4.90% respectively. Relative abundance graphs have been produced using scaled data presenting abundances across all datasets but grouped into each variable

The datasets represent various geographical locations, including China, Canada, Netherlands, France, Spain and the United Kingdom (UK). For simplicity when discussing the mainland European regions (France, Netherlands and Spain), they have been grouped together. Chinese datasets were the same Tibetan breed datasets discussed previously. Unknown genera were most abundant ranging from 28.48% (Canada) to 68.29% (UK) of total relative abundances (Fig. 6). *Lactobacillus* dominates in Canadian and European datasets (28.79% & 27.48% of total relative abundances), as *Streptococcus* and *Faecalibacterium* (10.54% & 7.21% of total relative abundances respectively) follow in abundance respectively. Meanwhile, UK datasets were more varied, with *Eisenbergiella* being most abundant, closely followed by *Oscillibacter* and *Faecalibacterium* (6.46%, 5.84% & 4.90% of total relative abundances respectively). *Ruminiclostridium* and *Butyricicoccus* were also more abundant in the UK (2.68% & 2.64% of total relative abundances) compared with Canadian and European datasets; with *Romboutsia* and *Enterococcus* (7.41% & 6.42% of total relative abundances) being abundant in Canadian dataset, and *Alistipes* and *Bacteroides* (3.52% & 3.50% of total relative abundances) being abundant in European datasets. Conversely, *Bacteroides* and *Parabacteroides* dominate Chinese datasets (28.08% & 1.62% of total relative abundances). Overall, the median diversity is similar across each location, although UK chicken datasets were the most diverse closely followed by Canada, likewise in terms of evenness. Meanwhile Canadian and Chinese datasets present the most richness across all datasets; significant differences were observed across each alpha diversity index examined (*P* ≤ 0.001) (Supplementary Fig. 4; Table 2).

### Common core microbiome

**Fig. 7:**
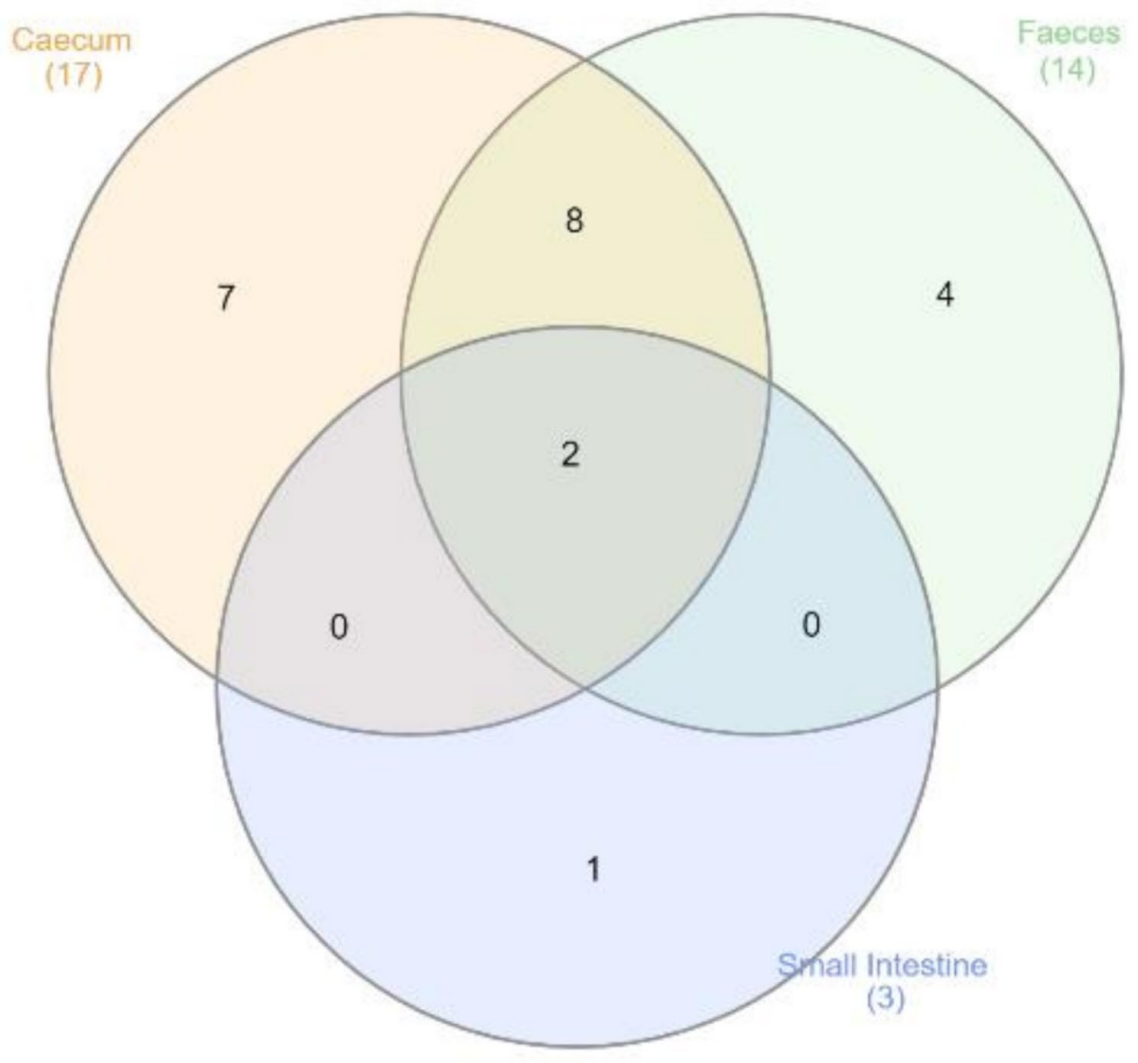
Number of genera present amongst each GIT site as common core members. Venn diagram showing the number of genera identified as being common core across the caecum, faeces and small intestine datasets irrespective of variables. The graph highlights two genera (*Faecalibacterium* & *Lactobacillus*) were shared between each GIT site datasets. Graph was produced using the website http://www.interactivenn.net/index.html [20]

Only *Faecalibacterium* and *Lactobacillus* were identified as common core microbiota members, across all GIT sites (Table 3; Fig. 7). Seventeen genera were identified as caecum common core microbiome, including *Faecalibacterium*, *Lactobacillus*, *Blautia*, *Butyricicoccus* and *Eisenbergiella*. Likewise, 14 genera were identified as members of the common core microbiome of faeces, including *Enterococcus*, *Lactobacillus*, *Streptococcus*, *Clostridium*, *Blautia*, *Erysipelatoclostridium*, *Faecalibacterium* and *Butyricicoccus*. Amongst the small intestine, five and four genera respectively were identified as members of the core microbiome of the ileum and jejunum and only *Faecalibacterium*, *Lactobacillus* and *Microbacterium* were common in both small intestine regions (Table 3; Fig. 7). In conclusion, 17, 14 and 3 genera were identified as members of the common core microbiome across datasets associated with the caecum, faeces and small intestine respectively, as 8 genera were common in the caecal and faecal communities, where *Faecalibacterium* and *Lactobacillus* were common across each GIT site (Fig. 7).

**Table 3:** Members of the common core microbiome across each GIT site at Genus level. Listed below were the common core genera present across > 90% of datasets associated with each GIT site

Caecal	Faecal	Small intestine
*Anaerostipes*	*Blautia*	*Faecalibacterium*
*Anaerotruncus*	*Butyricicoccus*	*Lactobacillus*
*Blautia*	*Clostridium*	*Microbacterium*
*Butyricicoccus*	*Eisenbergiella*	
*Clostridium*	*Enterococcus*	
*Eisenbergiella*	*Erysipelatoclostridium*	
*Erysipelatoclostridium*	*Faecalibacterium*	
*Faecalibacterium*	*Lachnoclostridium*	
*Fusicatenibacter*	*Lactobacillus*	
*Intestinimonas*	*Paucibacter*	
*Lachnoclostridium*	*Romboutsia*	
*Lactobacillus*	*Roseburia*	
*Negativibacillus*	*Streptococcus*	
*Oscillibacter*	*Subdoligranulum*	
*Roseburia*		
*Ruminiclostridium*		
*Subdoligranulum*		

## Discussion

Our aim was to identify the overall and ‘core’ gastrointestinal tract (GIT) microbiota of broiler chickens and assess the effects of GIT site, breed, age and geographical location on the microbial diversity, using publicly available data from the Sequence Read Archive (SRA) database. A total of 602 sequence datasets pertaining to 16S rRNA gene V3-V4 hypervariable regions were distributed into groups according to the metadata provided (GIT site, breed and age of birds as well as their geographical origin).

Firmicutes dominated followed by Proteobacteria and Bacteroidetes; *Ruminococcaceae*, *Lactobacillaceae* and *Lachnospiraceae* predominated composing half of all sequencing reads. At genus level most reads were unknown, although *Lactobacillus* is most abundant of those identified, followed by *Faecalibacterium* and *Eisenbergiella*. Of which, we suggest *Lactobacillus*, *Faecalibacterium*, *Butyricicoccus*, *Eisenbergiella*, *Subdoligranulum*, *Oscillibacter*, *Clostridium* & *Blautia* the common core microbiome of the gastrointestinal tract. These genera have been described as commensal and beneficial to broilers, aiding in producing short chain fatty acids (SCFA) from non-starch polysaccharides (NSP), alongside contributing to immune response regulation and overall benefiting bird’s health [24–29]. Analysis by PERMANOVA suggests that each variable considered in this study had a significant impact on microbiota diversity. However, it is worth noting that dietary differences across the samples, a well-established factor influencing microbiota diversity, constitute a confounder in the present study, requiring careful interpretation of the results. This limitation could have influenced not only the residual variance observed in the PERMANOVA results (R^2^ = 0.254) but also the variance attributed to the variables included in the model, potentially leading to an overestimation of their effects. Nevertheless, these findings provide valuable insights into the broader patterns and key determinants of microbiota diversity, offering a foundation for future research aimed at identifying consistent microbial signatures across diverse conditions.

### Gastrointestinal tract site

When discussing the most prominent genera per GIT site (small intestine, caeca and faeces), microbial communities differ dependent on function [30]. The small intestine facilitates digestate transportation to the caeca, evident by reduced enzymatic activity; while the caeca’s anaerobic environment facilitates nitrogen recycling, producing SCFAs from NSPs [30, 31]. Otherwise, the large intestine is short and does not retain digestate for an extensive period of time, but is involved with nutrient absorption [30]. The nature of faecal communities, as an ethically viable approach as a reference point for quantifying and identifying most members of the gut microbiota [32, 33], is reflected in our results as microbial communities from the small intestine (jejunum and ileum) and faeces resemble each other most. Of course, faecal community should not be reported as a ‘true’ representation of the intestinal or caecal microbiota [34, 35]. This is in contrast to Richards-Rios et al. 2020 [36] who highlight similarity in the caecal and ileal microbiota, especially during the earlier days of a broiler’s life. Alpha diversities also suggest differences, faecal communities have greater richness, as the caecal community is most even and is most diverse, closely followed by faecal. Diversity, furthermore, was reported by Wei et al. 2013 [17].

Excluding the caecal community, *Lactobacillus* is most prominent in each GIT; as *Candidatus arthromitus* and *Faecalibacterium* were amongst the most abundant amongst the ileum, *Faecalibacterium* and *Stenotrophomonas* in the jejunum as the following genera. *Stenotrophomonas* is an environmental bacteria commonly found in water, a bacterium which is rapidly emerging as a multi-drug resistant pathogen [37]. Meanwhile, *Streptococcus*, *Enterococcus* and *Romboutsia* follow in the faecal community, each of which were highly abundant amongst the chicken GIT microbiota [50]. *Faecalibacterium*, *Eisenbergiella* and *Oscillibacter* were most prominent in the caecal community. In doing so, the caeca present greatest levels of unclassified, or ‘unknown’, genera (59.58% of total abundances), compared to the remaining GIT sites (reaching 32.83% of total abundances). As the small intestinal and caecal microbiomes differ, previous indications suggest there should be some similarity due to the passage of contents and excreta [30].

Using definitions of the common core microbiome by Chica Cardenas et al. 2021 [18], *Lactobacillus*, *Faecalibacterium*, *Butyricicoccus*, *Eisenbergiella*, *Subdoligranulum*, *Erysipelatoclostridium*, *Lachnoclostridium*, *Roseburia*, *Clostridium* & *Blautia* were identified, being present across 90% of datasets in caecal and faecal communities, each of which have been previously reported [33]; only *Faecalibacterium* and *Lactobacillus* were present across 90% of datasets in each GIT site (caecal, faecal and small intestine). These were commonly identified amongst publications investigating the GIT microbiome, for instance, *Clostridium*, *Roseburia* and *Blautia* are capable of producing butyrate from widely accessible metabolites [51], the SCFA butyrate is recognised as one of the most efficient compounds in nutrient provision to the host [52]. Likewise, these common community members are highly involved with producing SCFAs from complex polysaccharides [53], these in turn provide accessible nutrients for the host for growth and other functions [38].

Our small intestine and caecal communities present differences from Wei et al. 2013 [17] as we see *Clostridium* and *Ruminococcus* as dominating within the ‘intestinal microbiome’, followed by *Lactobacillus*, *Bacteroides*, *Faecalibacterium* and *Eubacterium*. The jejunum and ileum are expected to be similar, as is seen in our results, due to their proximity and related functions [30]. *Ruminococcus*, *Clostridium* and *Eubacterium* were most abundant amongst the caecal community according to Wei et al. 2013 [17], which differs from our results which resemble those found be Chica Cardenas et al. 2021 [18] more as they also show that Gram-positive cocci, *Bifidobacterium*, *Clostridium*, *E. coli*, *Lactobacillus*, *Streptococcus*, *Bacteroidetes* were present. Of which, they also show that *Oscillospira*, *Lactobacillus*, *Faecalibacterium*, *Clostridium* and *Ruminococcus* were deemed as core microbiome members [18], which is what we note with the exception of *Clostridium*. Nonetheless, like our results, Wei et al. 2013 [17] identified the caeca as containing the most diverse communities. Meanwhile, our faecal community resemble the data published by Videnska et al. (2014), being comprised of Firmicutes, Proteobacteria, Bacteroidetes and Actinobacteria (76.2%, 14%, 6.5% & 3.8% respectively). Furthermore, Hou et al. 2016 [39] identified *Clostridium* (23.44%), *Bacteroides* (18.78%), *Lactobacillus* (8.77%), *Ruminococcus* (3.97%), *Hallella* (1.61%), *Subdoligranulum* (1.07%), *Faecalibacterium* (1.04%), *Roseburia* (0.98%), and *Eubacterium* (0.31%) as the common core, matching our global data.

### Breed

With regards to the GIT microbial communities between breeds, we identified similarities, whereby the main commercial breeds, AIL F8 Progeny, Cobb 500 and Ross 308, present *Lactobacillus* as the most abundant; microbial community members vary beyond this, with *Faecalibacterium* as next prominent in AIL F8 progeny, *Streptococcus* in Cobb 500, *Eisenbergiella*, *Oscillibacter* and *Faecalibacterium* in Ross 308, denoting these breeds still present similarities, despite undergoing different rearing methodology. Sasso T451A and Tibetan breeds represent non-commercial breeds with, the former as most diverse, followed by the Tibetan breeds; AIL F8 progeny and Cobb 500 had similar diversity, with as the Ross 308 breed was least diverse. Other publications also suggest breed influences the overall GIT microbiome, for example Pandit et al. (2018) noted a domination of Bacteroidetes/Firmicutes in Ross 308 and Kadaknath breeds, as opposed to Cobb 400 and Aseel breeds whereby caecal bacteria communities had an overall combined abundance ranging between 76.6% and 90.8%. Ghagus and Nicobari breeds also present Bacteroidetes/Firmicutes as most abundant [40].

### Bird age

As animals age, the GIT microbiota develops due to changing metabolic function, immune interactions, feed introduction, environmental changes amongst other factors [10]. Our findings suggest a development of the microbiota by increased of diversity and development of common genera. We note that *Lactobacillus* dominates during 1 week of age and 4 weeks of age, although, the microbiome stabilises with age to contain *Streptococcus*, *Eisenbergiella*, *Oscillibacter*, *Faecalibacterium*, *Butyricicoccus*, *Subdoligranulum*, *Faecalibacterium* and *Bacteroides*. Eventually a combination of *Bacteroides*, *Megamonas*, *Faecalibacterium*, *Eisenbergiella*, *Alistipes*, *Lactobacillus*, *Methanocorpusculum* and *Parabacteroides* were most prominent during the latter ages of this study. This is indicative of previous publications, which show that bacteria were present upon hatch and undergo successional development where the bacterial taxa stabilise with age [8, 10, 41]. For example, Glendinning et al. 2019 [42] note the development from a *Clostridium sensu stricto 1* dominating community within the cereal and intestinal communities during the earlier ages, towards a community containing *Enterococcus*, *Escherichia/Shigella* and *Lactobacillus*, with age. Xiao et al. 2021 [43] identify *Escherichia* and *Clostridium* as the dominating genera at day 0 in laying hens, developing towards *Lactobacillus* domination, finally to a community persisting of *Bacteroides*, *Odoribacter* and *Clostridiales vadin BB60* group by day 50. Importantly, like our global data, Glendinning et al. 2019 [42] and Xiao et al. 2021 [43] both highlight the increase of diversity as the birds age.

### Geographical location

It is evident that the microbial composition of GIT microbiota from different locations is highly varied [8, 10, 41]. This as further environmental factors, both abiotic (elevation, temperature, humidity, water) and biotic (predators and prey), and dietary differences, all of which play a significant role in influencing the microbiome. *Lactobacillus* was similar in abundance across both the Canadian and European datasets, as *Eisenbergiella* is most abundant in the UK, with only the Chinese datasets showing a significant abundance of *Bacteroides* (although a potential study-specific factor). The variation amongst geographical locations is recorded by Pin Viso et al. 2021 [44] who highlight a high of variation amongst regions included in their study: Argentina, Australia, Croatia, Germany, Hungary, Malaysia and the United States. They highlight *Lactobacillaceae* dominated within European countries (Croatia, Germany, Hungary and Slovakia), except for Germany where *Bacteroidaceae* dominated [44]. There was high variation of *Bacteroidaceae*, *Lactobacillaceae*, *Lachnospiraceae*, *Ruminococcaceae* and *Clostridiaceae* [44]. Pandit et al. 2018 [45] compared caecal microbiota of Aseel and Kadaknath breeds sourced from two separate farms and identified that both breed and geographical locations affect GIT bacterial diversity. They found that *Bacteroides* dominated in both locations (> 20% of total abundances), except for Kadaknath breeds at farm 2 where *Fusobacteria* dominated (44.1% of total abundances). Farm 1 showed that the Aseel breed were subsequently dominated by *Clostridium* (6.5%), whilst *Alistipes* (23.1% of total abundances) followed in abundance in the Kadaknath bred; this differs at Farm 2 as Aseel breeds on this farm had *Alistipes* as the second most abundant GIT bacteria (6.4% of total abundances), while the Kadaknath breed had *Bacteroides* as the second most abundant GIT bacteria (21.6% of total abundances) [45]. Ultimately this highlights that there are environmental impacts which have a role in shaping the GIT microbiome.

## Conclusion

The aim of this study was to identify the core microbiota of a chicken gastrointestinal tract using data made available through the SRA database and to assess the effect of key variables - GIT site, breed, age and geographical location - on gastrointestinal tract microbial diversity. We identified significant influences by each variable examined including the GIT site, geographical location, breed and age of birds, regardless of dietary factors. It was apparent that irrespective of the considered evalauted key factor, *Lactobacillus* is the prominent genus of the chicken GIT bacterial community. Identifying the common core microbiome of the caecal and faecal communities suggest *Lactobacillus*, *Faecalibacterium*, *Butyricicoccus*, *Eisenbergiella*, *Subdoligranulum*, *Oscillibacter*, *Clostridium* & *Blautia* are part of the core chicken GIT microbiome, meanwhile, only *Faecalibacterium* and *Lactobacillus* were present across 90% of datasets for all evaluated factors associated with the GIT. In terms of breed, differences in the microbial communities were found with Ross 308, Cobb 500 and Sasso T451A having the most diverse bacterial GIT communities although unaccounted factors, such as diet, could also contribute to these results. Furthermore, there was clear evidence of a successional development in the microbial community as the birds age, with the caecal microbial communities transitioning from a *Lactobacillus* dominated community to a stabilised community dominated by *Faecalibacterium*, *Eisenbergiella*, *Bacteroides*, *Megamonas*, and *Lactobacillus*. Meanwhile, geographical location also impacts the community, as *Eisenbergiella* and *Bacteroides* were dominate amongst China and UK, while *Lactobacillus* dominates in both the Canadian and European datasets. This study provided an estimative of what ‘normal’ is within poultry GIT microbiota globally, alongside defining the key factors which affect the diversity present, which is imperative to enhancing the microbiome for productive and environmental improvements.

## Electronic supplementary material

Below is the link to the electronic supplementary material.

Supplementary Material 1

Supplementary Material 2

Supplementary Material 3

## Data Availability

The raw sequence data reported in this paper were already publicly available as noted in supplementary Table 1.
