# Supplementary material for: Decoding the chicken gastrointestinal microbiome
Source: BMC Microbiol. 2025 Jan 20;25:35. doi: 10.1186/s12866-024-03690-x (PMC11744950; doi:10.1186/s12866-024-03690-x)
Supplement: Supplementary file 3 — Supplementary Material 3 [file 12866_2024_3690_MOESM3_ESM.docx]

## **Supplementary Material**


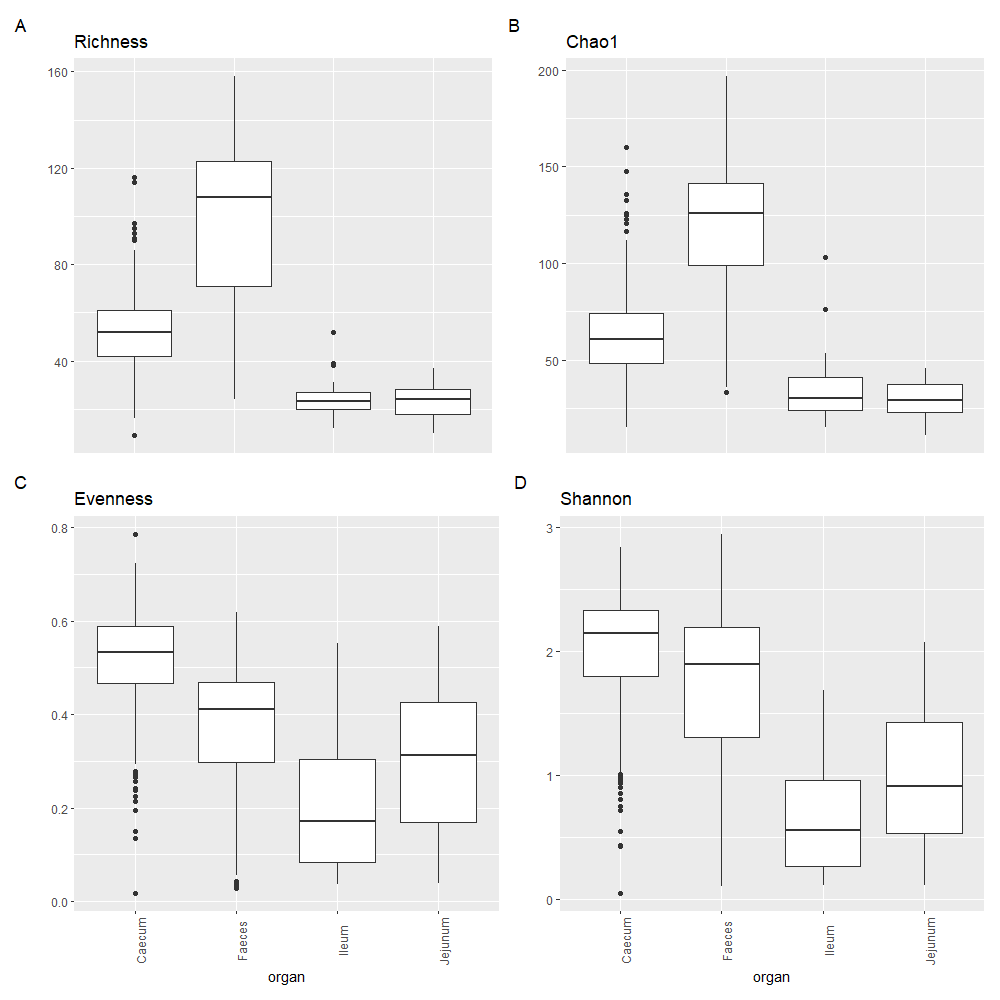


**Supplementary Figure 1. Boxplots describing alpha diversity indices of the GIT locations amongst datasets.** Four separate plots depict the richness, Chao1, evenness and shannon diversity of each GIT region. **A**. Presents the richness of each GIT region where faeces have been identified as most rich, followed by caecum, as the small intestine regions are similar; **B**. Presents the Chao1 richness of each GIT region where faeces have been identified as most rich, followed by caecum, as the small intestine regions are similar; **C**. Presents the evenness of each GIT region where the caecum have been identified as most even, followed by faeces, as the small intestine regions are similar; **D**. Presents the Shannon diversity of each GIT region where the caecum have been identified as most diverse closely followed by faeces, meanwhile the small intestine regions are similar with jejunum slightly more diverse. Plots were produced in R using the ‘vegan’ package (ver. 2.4-6).


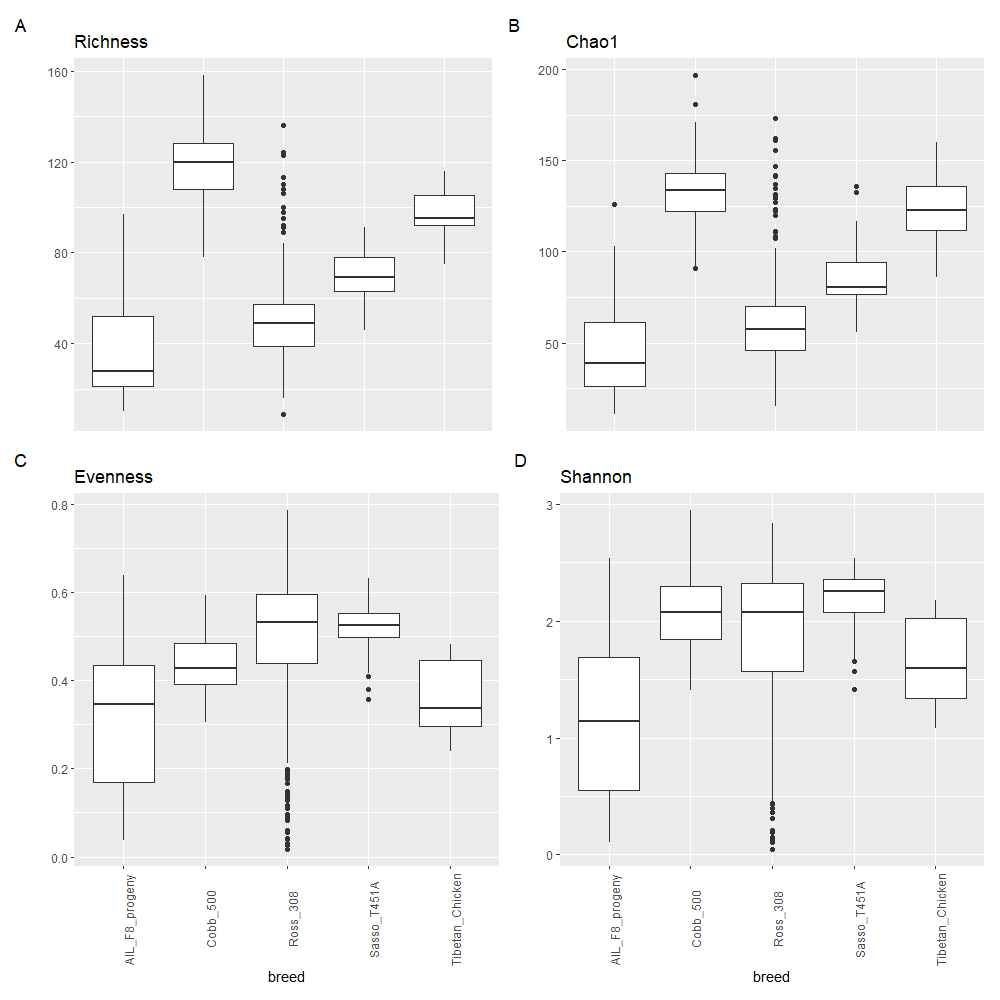


**Supplementary Figure 2. Boxplots describing alpha diversity indices of breeds amongst datasets.** Four separate plots depict the richness, Chao1, evenness and shannon diversity of each breed. **A**. Presents the richness of each breed where Cobb 500 have been identified as most rich, followed by the Tibetan chicken breeds, Sasso T451A and Ross 308, as AIL F8 is least rich; **B**. Presents the Chao1 richness of each breed where Cobb 500 have been identified as most rich, followed by the Tibetan chicken breeds, Sasso T451A and Ross 308, as AIL F8 is least rich ; **C**. Presents the evenness of each breed where the Ross 308 and Sasso T451A have been identified as most even, followed by Cobb 500 then the Tibetan chicken breeds, as AIL F8 as least; **D**. Presents the Shannon diversity of each breed where the Cobb 500, Ross 308 and Sasso T451A have been identified as most diverse closely followed by the Tibetan chicken breeds, as AIL F8 is least diverse. Plots were produced in R using the ‘vegan’ package (ver. 2.4-6).


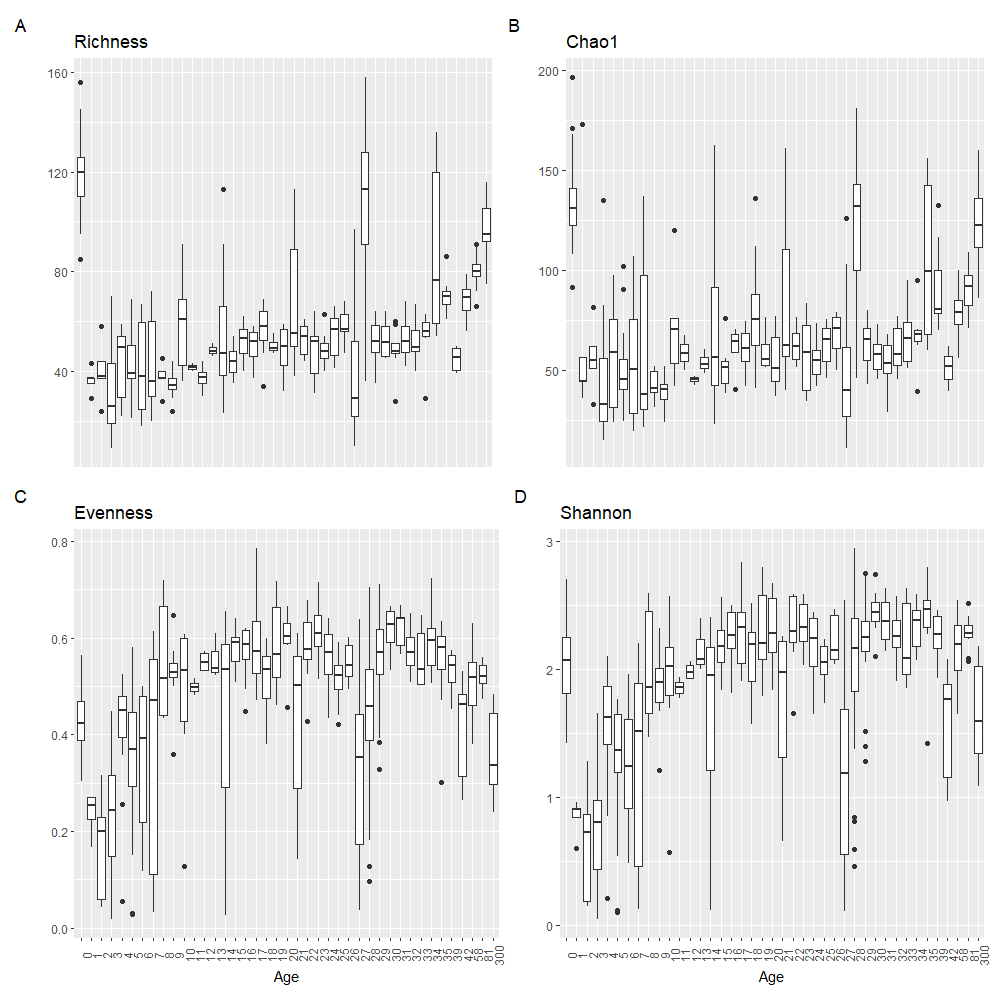


**Supplementary Figure 3. Boxplots describing alpha diversity indices of age amongst datasets.** Four separate plots depict the richness, Chao1, evenness and shannon diversity of each age. **A**. Presents the richness of each age where days 0 and 27 have been identified as most rich, as the remaining progressing ages gradually increase; **B**. Presents the Chao1 richness of each age where days 0 and 27 have been identified as most rich, as the remaining progressing ages gradually increase; **C**. Presents the evenness of each age where the evenness increases overtime, with a slight decrease from day 30 onwards; **D**. Presents the Shannon diversity of each age where diversity increases over time. Plots were produced in R using the ‘vegan’ package (ver. 2.4-6).


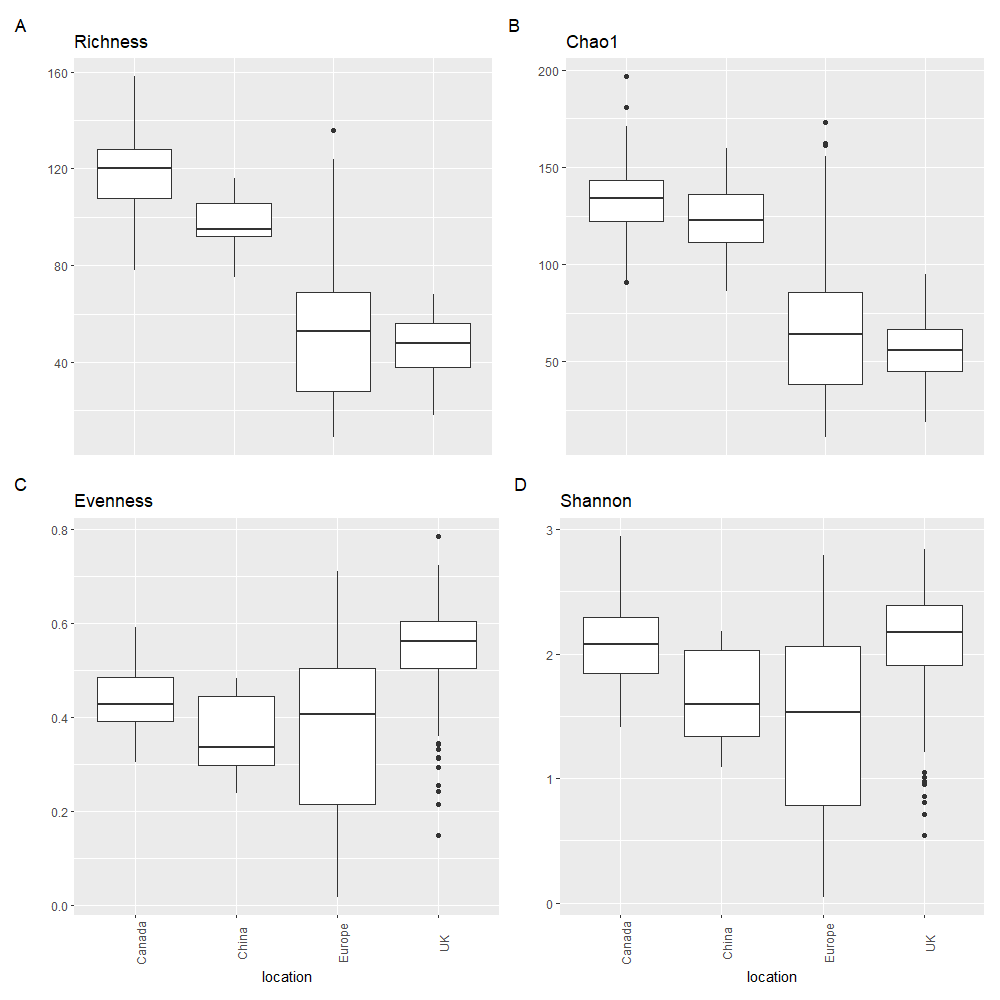


**Supplementary Figure 4. Boxplots describing alpha diversity indices of geographic locations amongst datasets.** Four separate plots depict the richness, Chao1, evenness and shannon diversity of each GIT region. **A**. Presents the richness of each geographical location where Canadian datasets, closely followed by Chinese, have been identified as most rich, as European and UK datasets are similar; **B**. Presents the Chao1 richness of each geographical location where Canadian datasets, closely followed by Chinese, have been identified as most rich, as European and UK datasets are similar; **C**. Presents the evenness of each geographical location where the UK datasets have been identified as most even, where the remaining locations are similar, albeit, vary in range; **D**. Presents the Shannon diversity of each geographical location where the UK and Canadian datasets have been identified as most diverse followed by the Chinese and European datasets which are similar, although vary in range. Plots were produced in R using the ‘vegan’ package (ver. 2.4-6).

**Supplementary Table 1. List of bioprojects, associated studies and number of datasets per bioproject and study parameters.** Each study was carried out between 2016 and 2020, equating to 610 datasets. These would further be refined removing 8 datasets (4 from Ijaz *et al.* (2018) and 4 from Borey *et al.* (2020)) to meet our criteria mentioned in ‘Selection of SRA data and bioprojects’.

| **BioProject** | **Author** | **Trimmed** | **F Primer** | **R Primer** | **No Datasets** | **Organ** | **Breed** | **Age** | **Broiler/Hen** | **Gender** | **Geographical Location** | **Diet** |
| --- | --- | --- | --- | --- | --- | --- | --- | --- | --- | --- | --- | --- |
| PRJEB25776 | 46 | Yes | 27F 5’TCGTCGGCAGCGTCAGATGTGTATAAGAGACAGCCTACGGGNGGCWGCAG 3’ | 805R 5’GTCTCGTGGGCTCGGAGATGTGTATAAGAGACAGGACTACHVGGGTATCTAATCC 3’ | 247 | Caecal | Ross 308 | 1-35 days | Broiler | Male | Northern Ireland - UK | Commercial Diet |
| PRJNA296553 | 23 | Yes | 341F  (5′- ATTACCGCGGCTGCTGG- 3′) | 534R with GC clamps  (5′- CGCCCGCCGCGCGCGGCG GGCGGGGCGGGGGCACGGGGGGCCTA CGGGAGGCAG CAG- 3′) | 2 | Caecal | LS Tibetan Chicken | >300 days | Broiler | Female | China – Tibet | Free Range |
| PRJNA299358 | 23 |  |  |  | 1 | Caecal | GZ Tibetan Chicken | >300 days | Broiler | Female | China – Sichuan |  |
| PRJNA299519 | 23 |  |  |  | 1 | Caecal | QH Tibetan Chicken | >300 days | Broiler | Female | China – Qinghai |  |
| PRJNA299520 | 23 |  |  |  | 1 | Caecal | DQ Tibetan Chicken | >300 days | Broiler | Female | China – Yunnan |  |
| PRJNA299524 | 23 |  |  |  | 1 | Caecal | Lohmann Laying Hen | >300 days | Laying Hen | Female | China – Sichuan |  |
| PRJNA299526 | 23 |  |  |  | 1 | Caecal | Daheng Broilers | >300 days | Broiler | Female | China – Sichuan |  |
| PRJEB29068 | 8 | Yes | 515F 5’GTGCCAGCMGCCCGCGGTAA 3’ | 806R 5’ GGACTACHVGGGTWTCTAAT 3’ | 63 | Caecal | Sasso-T451A | 0-42 days | Slow growing meat chicken | N/A | Spain – Bilbao | Free Range |
| PRJEB29068 | 8 |  |  |  | 20 | Caecal | Ross 308 | 0-12 weeks | Broiler | N/A | Spain – Bilbao | Commercial Diet |
| PRJNA611508 | 47 | Yes | PCR1F_343 5’-CTTTCCCTACACGACGCTCTTCCGATCTACG GRAGGCAGCAG-3’ | PCR1R_784 5’-GGAGTTCAGACGTGTGCTCTTCCGATCTTAC CAGGGTATCTAATCCT-3’ | 38 | Caecal | AIL F8 Progeny | 0-27 days | Broiler | Both | France – Nouzilly | Low Digestibility |
| PRJNA611508 | 47 |  |  |  | 38 | Ileal | AIL F8 Progeny | 0-27 days | Broiler | Both | France – Nouzilly | Low Digestibility |
| PRJNA611508 | 47 |  |  |  | 37 | Jejunum | AIL F8 Progeny | 0-27 days | Broiler | Both | France – Nouzilly | Low Digestibility |
| PRJNA517082 | 48 | Yes | CVI_V3-forw CCTACGGGAGGCAGCAG | CVI_V4-rev GGACTACHVGGGTWTCT | 60 | Faecal | Ross 308 | 0-35 days | Broiler | Male | Netherlands | Commercial Diet |
| PRJNA602334 | 49 | Yes | 515FP1-CS1F ACACTGACGACATGG TTCTACAGTGCCAGCMGCCGCGGTAA | 806RP1-CS2R TACGGTAGCAGAGACTTGGTCTGGACTACHVGGGTWTC TAAT | 92 | Faecal | Cobb 500 | 28-49 weeks | Hen | Female | Canada - Quebec | N/A |

**Supplementary Table 2. Total number of OTU divided into GIT section per breed, geographical location and age. A**: Counts of available datasets from variables grouped as per GIT section, breed and geographic location; **B**: Counts of available datasets from variables grouped as per GIT section age. Each group contain 602 total datasets.

**A.**

| **Variable** | ***n=*** | **Variable** | ***n=*** | **Variable** | ***n=*** |
| --- | --- | --- | --- | --- | --- |
| Caecum | 375 | Caecum AIL F8 progeny | 38 | Caecum UK | 247 |
| Faecal | 152 | Caecum Ross 308 | 267 | Caecum Europe | 121 |
| Ileum | 38 | Caecum Sasso T451A | 63 | Caecum China | 7 |
| Jejunum | 37 | Caecum Tibetan Chicken | 7 | Faeces Canada | 92 |
|  |  | Faeces Ross 308 | 60 | Faeces Europe | 60 |
|  |  | Faeces Cobb 500 | 92 | Ileum Europe | 38 |
|  |  | Ileum AIL F8 progeny | 38 | Jejunum Europe | 37 |
|  |  | Jejunum AIL F8 progeny | 37 |  |  |

**B.**

| **Variable** | ***n=*** | **Variable** | ***n=*** | **Variable** | ***n=*** | **Variable** | ***n=*** |
| --- | --- | --- | --- | --- | --- | --- | --- |
| Faeces 0 days old | 46 | Caecum 9 days old | 8 | Faeces 21 days old | 5 | Caecum 31 days old | 9 |
| Faeces 1 days old | 5 | Faeces 10 days old | 5 | Caecum 21 days old | 8 | Caecum 32 days old | 8 |
| Faeces 2 days old | 5 | Caecum 10 days old | 3 | Caecum 22 days old | 12 | Caecum 33 days old | 6 |
| Faeces 3 days old | 5 | Caecum 11 days old | 2 | Caecum 23 days old | 11 | Caecum 34 days old | 7 |
| Caecum 3 days old | 12 | Caecum 12 days old | 4 | Caecum 24 days old | 9 | Faeces 35 days old | 5 |
| Faeces 4 days old | 5 | Caecum 13 days old | 3 | Caecum 25 days old | 8 | Caecum 35 days old | 5 |
| Caecum 4 days old | 13 | Faeces 14 days old | 5 | Caecum 26 days old | 9 | Caecum 39 days old | 12 |
| Faeces 5 days old | 5 | Caecum 14 days old | 10 | Jejunum 27 days old | 37 | Caecum 42 days old | 6 |
| Caecum 5 days old | 9 | Caecum 15 days old | 7 | Caecum 27 days old | 42 | Caecum 58 days old | 16 |
| Faeces 6 days old | 5 | Caecum 16 days old | 9 | Ileum 27 days old | 38 | Caecum 81 days old | 17 |
| Caecum 6 days old | 7 | Caecum 17 days old | 11 | Faeces 28 days old | 51 | Caecum 300 days old | 7 |
| Faeces 7 days old | 5 | Caecum 18 days old | 20 | Caecum 28 days old | 10 |  |  |
| Caecum 7 days old | 8 | Caecum 19 days old | 10 | Caecum 29 days old | 16 |  |  |
| Caecum 8 days old | 5 | Caecum 20 days old | 8 | Caecum 30 days old | 8 |  |  |

**Supplementary File 1. Microsoft Excel Document.** Data and metadata relating to the datasets utilised in this study on phylum, family and genus taxonomic levels. ‘**Supplementary File 1A. Excel (Raw Data)**’ contains raw taxonomic data; relative abundances have been calculated without any additional grouping into variables. ‘**Supplementary File 1B. Excel (Variable Data)**’ contains grouped data; relative abundances from previous have been grouped together to obtain the relative abundance of variables.

**Supplementary File 2. R Script Document.** Script produced in R for processing statistical analysis on data including ANOVA, Alpha Diversity, Beta Diversity amongst others.
